# The Usefulness of Deep Tendon Reflexes in the Diagnosis of Lumbar Spine Diseases: A Narrative Review

**DOI:** 10.7759/cureus.55772

**Published:** 2024-03-08

**Authors:** Tadatsugu Morimoto, Hirohito Hirata, Kazuyuki Watanabe, Kinshi Kato, Koji Otani, Masaaki Mawatari, Takuya Nikaido

**Affiliations:** 1 Department of Orthopedic Surgery, Faculty of Medicine, Saga University, Saga, JPN; 2 Department of Orthopedic Surgery, Fukushima Medical University School of Medicine, Fukushima, JPN

**Keywords:** achilles tendon reflex, patellar tendon reflex, narrative review, lumbar spine disease, diagnosis, deep tendon reflex

## Abstract

The deep tendon reflex (DTR) is a more objective indicator than sensory and muscle assessments for lumbar spine disorders. Further, unlike sensory and muscle assessments that require patient cooperation, the DTR can be assessed even in patients with impaired consciousness or cognition. Therefore, DTR assessment with a hammer is an essential neurological test for lumbar spinal diseases. However, despite the usefulness of DTR assessment, few reports have described the significance of increased, diminished, or absent deep lower extremity reflexes in lumbar spine diseases. This review outlines the history of DTR of the lower limbs and describes the techniques, evaluation, and interpretation of DTR for the diagnosis of lumbar spine diseases. The patellar tendon reflex (PTR) was the first parameter of lower extremity DTR identified to have clinical usefulness, followed by the Achilles tendon reflex (ATR), pathological reflexes (Babinski reflex), and reflex enhancement (Jendrassik maneuver). They have now become an integral part of clinical examination. To determine whether an increase or decrease in DTR is pathological, it is necessary to determine left-right differences, differences between the upper and lower extremities, and the overall balance of the limb. There are several critical limitations and pitfalls in interpreting DTRs for lumbar spine diseases. Attention should be paid to examiner and patient factors that make the DTR assessment less objective. When there is a discrepancy between clinical and imaging findings and the level of the lumbosacral nerve root disorder is difficult to diagnose, the presence of a lumbosacral transitional vertebra, nerve root malformation, or furcal nerve should be considered. In addition, assessing the DTR after the gait loading test and standing extension loading test, which induce lumbosacral neuropathy, will help provide a rationale for the diagnosis.

## Introduction and background

Despite modern advances in imaging technology, neurological examinations remain important in lumbar spine disease, and deep tendon reflex (DTR) assessment with a hammer is a mandatory test [1-3]. The DTR is a reflex contraction of muscles that is commonly triggered by striking a tendon with a hammer [3]. In general, reflexes are objective clinical tests because they require no input from the brain and are involuntary and repeatable responses to specific stimuli [2]. Therefore, a DTR assessment can be more objective than sensory or motor assessments and can be obtained even if the patient is unable to communicate due to impaired consciousness or cognitive impairment. DTR assessment is an essential technique in daily practice and is included in the education for medical students’ objective structured clinical examinations [4]. In lumbar spine disorders, neurological examinations, including DTR assessments, are crucial for diagnosing and determining treatment options. However, reports describing the significance of increased, diminished, or absent lower limb DTR in the examination of lumbar spine disorders are lacking. The key to DTR assessment is to compare the diagnosis of the disability level in the context of the symptoms identified in the interview and the assessment of sensory and muscular strength. To determine whether an increase or decrease in DTR is pathological, it is necessary to evaluate the difference between the left and right extremities, the upper and lower extremities, and the balance of the entire extremities. Thus, the DTR responses of the lower limbs cannot be better interpreted without understanding the basic principles and key features of DTR. Importantly, clinicians need to learn the correct technique because the technique of eliciting DTR provides valuable information about the state of the nervous system [3].

The purpose of this review was to outline the history of DTR in the lower extremity and provide an account of the techniques, assessment and interpretation, and utility of DTR in the diagnosis of lumbar spine disease. In the Review section, a brief history of the essential hammers in DTR is provided. An overview of the patellar tendon reflex (PTR) and Achilles tendon reflex (ATR), their assessments (evaluation methods), pathological reflexes, and enhanced techniques for evoked potentiation used in lumbar spine diseases were also presented. The proper technique is critical for the validity of the findings. The Review section also describes the implications of enhanced, diminished, and absent lower limb DTR and provides additional knowledge that may be useful if there is any doubt about their interpretation. When there is a discrepancy between clinical and imaging findings and the level of lumbosacral nerve root disorder is difficult to diagnose, knowledge of lumbosacral transitional vertebrae, nerve root malformations, furcal nerve, and gait loading (a technique for inducing lumbosacral neuropathy) can contribute to rationalizing the diagnosis.

## Review

History of DTR and percussion hammer in lumbar spine diseases

The use of percussion for physical examination originated from winegrowers attempting to measure the volume of wine by tapping wine barrels [1,2,5]. The following is a summary of the history of hammering in the assessment of DTR, as summarized by several studies [1,2,5]. Percussions started to emerge in the late 18th century. In 1761, the Austrian physician Leopold Auenbrugger reported the clinical utility of percussion in the chest, back, and abdomen. Thereafter, the medical community began to acknowledge the usefulness of percussion in examinations. In 1875, Wilhelm Erb and Carl Westphal published a paper on DTR in the fifth volume, third issue of the “Archiv für Psychiatrie und Nervenkrankheiten” [1,2,5]. They reported, for the first time, the knee tendon reflex. Westphal confirmed that the PTR was still present even when the skin above the tendon was anesthetized [1,2,5]. This suggests that this reflex is not a skin reflex, although its significance was not initially understood. In 1877, Westphal presented to the Berlin Medical Society that the disappearance of the knee tendon reflex is an early symptom of tabes dorsalis, emphasizing that it is a crucial sign [1,2,5]. This was the first report on the diagnostic usefulness of the knee tendon reflex. Gowers also reported that the knee tendon reflex has entertained schoolchildren for generations, highlighting its unusual nature even in that era [1,2,5]. Gradually, its clinical significance has been elucidated, and it is now an indispensable factor in clinical examination. As seen in the logo of the American Neurological Association for the Taylor hammer, the observation of the tendon reflex has become a symbol of neurological examination. With the widespread adoption of tendon reflex examinations, percussion hammers have been used extensively. Subsequently, various reports suggested recommendations, such as striking the ulnar side of the palm (like a karate chop) or using percussion hammers. However, examinations using percussion hammers have become the standard. Disserol et al. [6] recently surveyed neurologists in Brazil and found that all respondent physicians used percussion hammers, and many preferred the Babinski-Rabiner hammer. Therefore, the hammer is a crucial item for neurologists.

The first percussion hammer was proposed by David Barry in 1828; however, the Taylor hammer, devised by John Madison Taylor, is considered the first hammer specifically designed for reflex detection [1]. Subsequently, prominent neurologists such as William Christopher Krauss, Bernhardt Berliner, and Joseph François Félix Babinski, among others, created and presented hammers based on their unique insights [1,2,5]. The hammers introduced here represent only a small fraction. Indeed, discovering the history of every hammer developed remains challenging; however, what is essential is not the form of the hammer but the fact that there was an absolute necessity for developing percussion tools, which proved the importance of DTR.

Evaluation of DTR in the lower extremity

In the evaluation of lower extremity DTR used in the examination of lumbar spine disease, it is vital to evaluate the PTR, Achilles tendon reflex (ATR), and pathological reflexes. In addition, DTR enhancement techniques are useful for accurate assessments.

PTR and ATR

**Figure 1:**
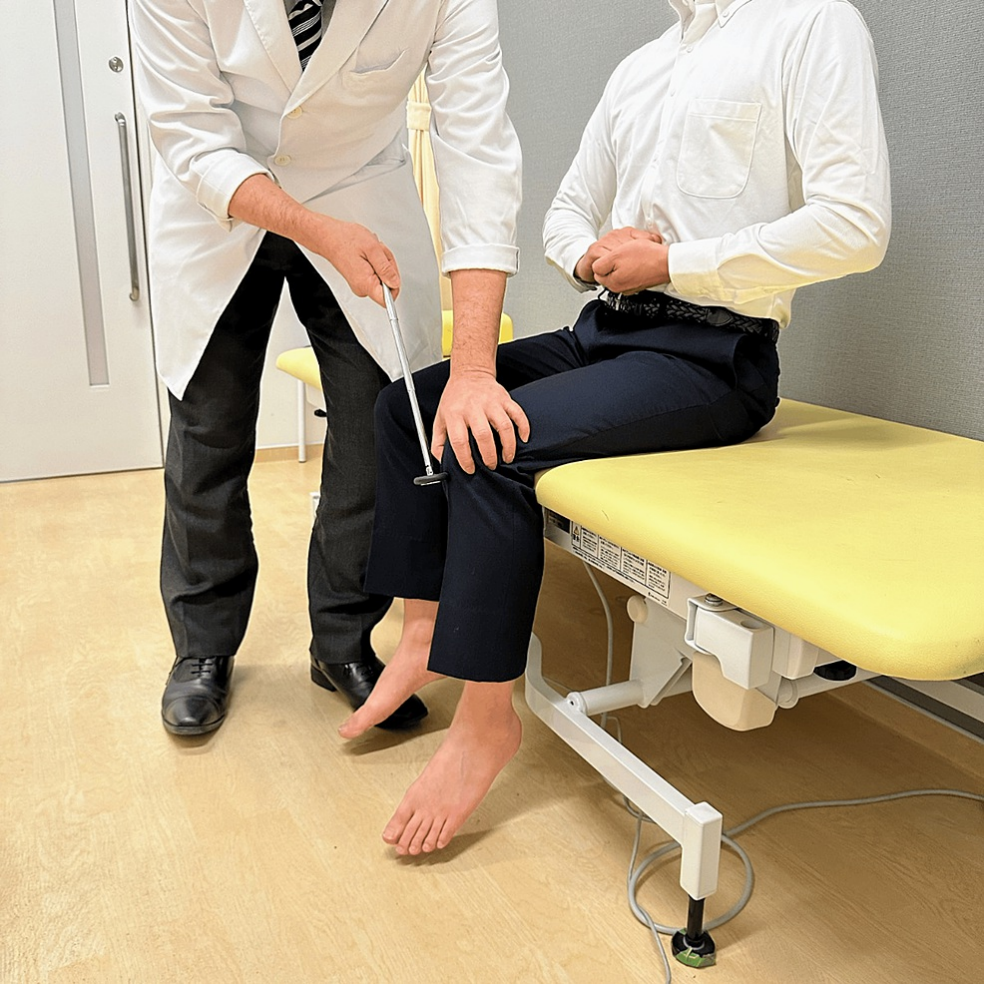
Patellar tendon reflex: sitting position, evaluation using the Jendrassik maneuver The legs should not touch the floor, and the knees should be at a 90° flexion. No reflexes should ever be described as “absent” unless the Jendrassik maneuver is performed.

**Figure 2:**
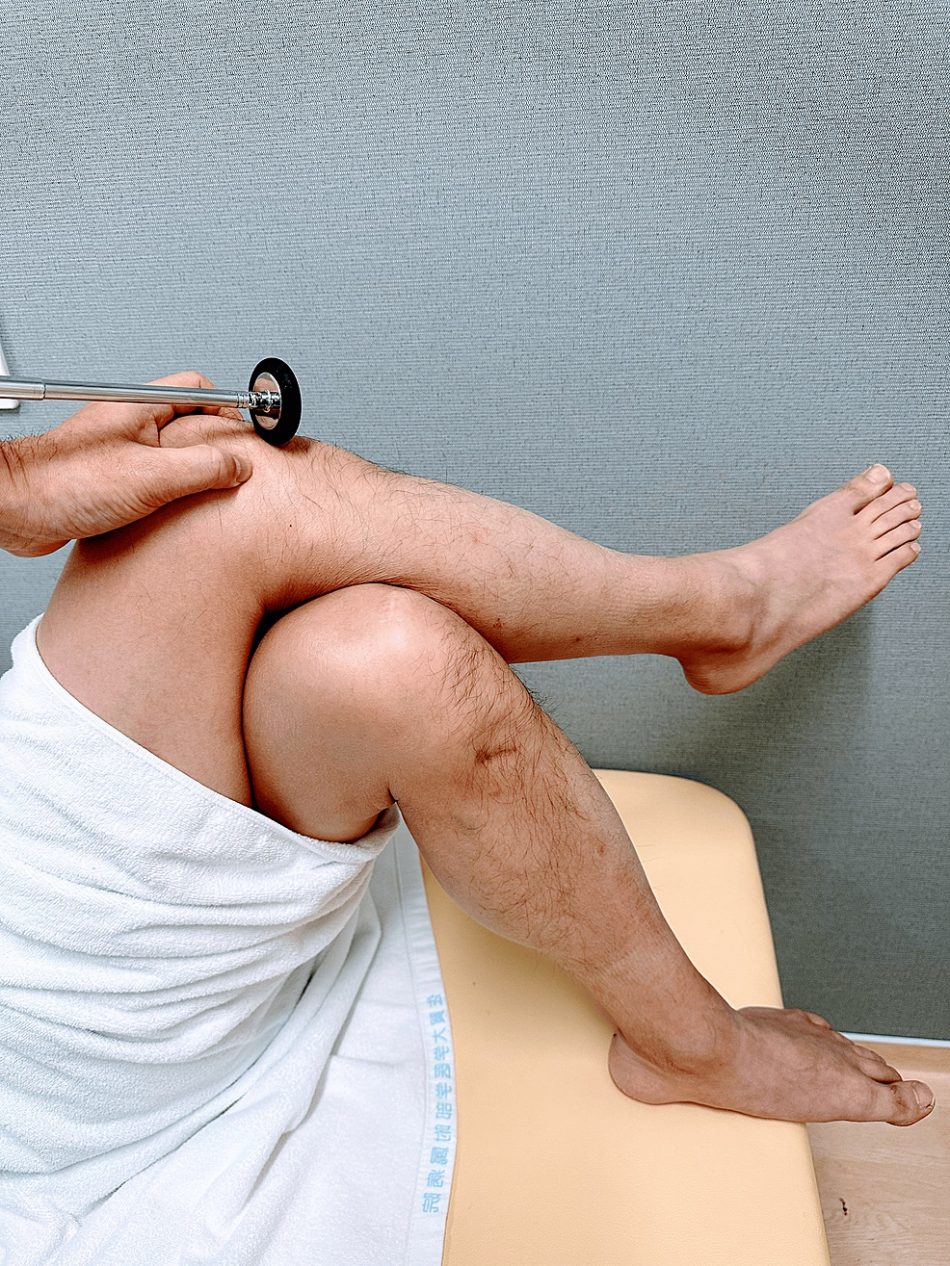
Patellar tendon reflex: supine position One leg is crossed over the other leg, and the uppermost leg is tapped.

Patellar tendon reflex (PTR) or knee jerk: The striking point for PTR is the patellar tendon, and myotome involves L3 and L4 innervating the quadriceps femoris muscle through the femoral nerve [3]. In the supine position, the knee and hip are flexed, and the examiner’s hands are placed under the knee. The entire lower extremity is relaxed, and the tendon is stretched. In the sitting position, the legs should not touch the floor, the knees should be at a 90° flexion (Figure 1) or one leg should cross over the other, and the upper leg should be tapped (Figure 2).

**Figure 3:**
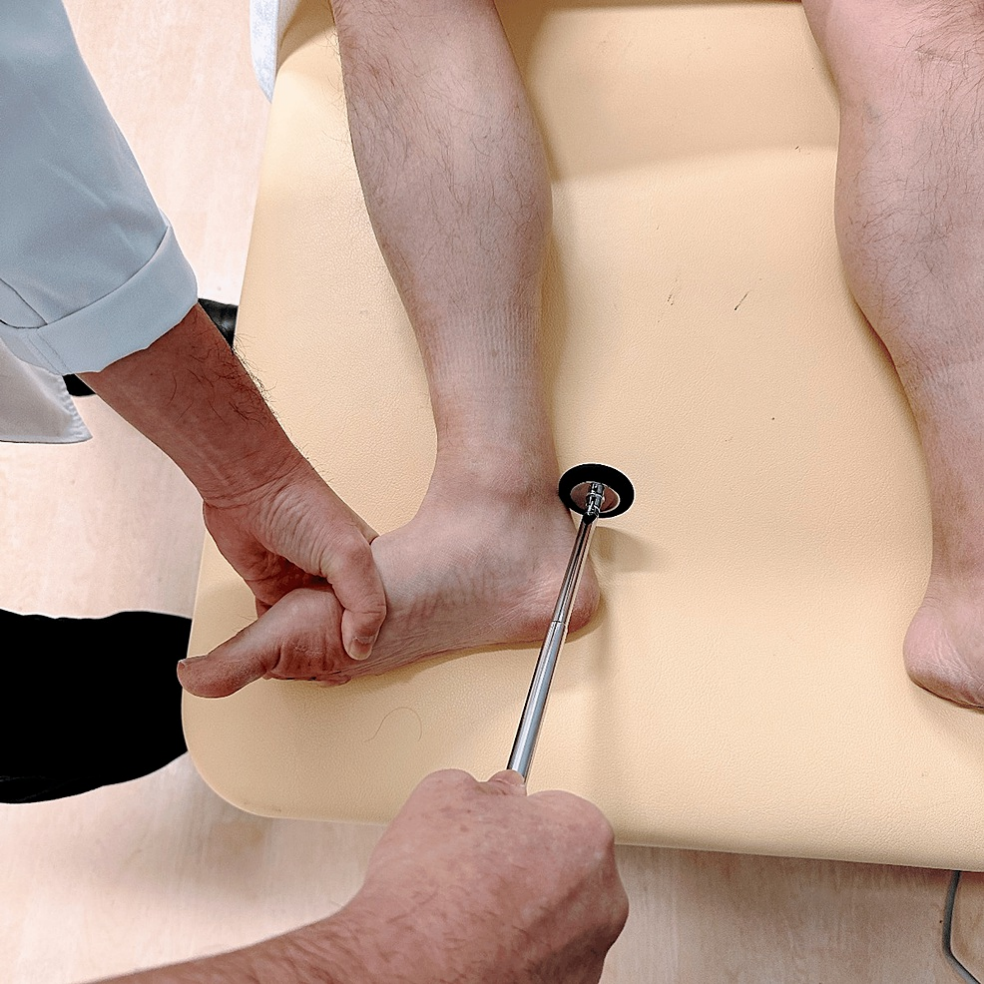
Achilles tendon reflex: supine position (frog-leg position) The knee is flexed with hip abduction and external rotation.

**Figure 4:**
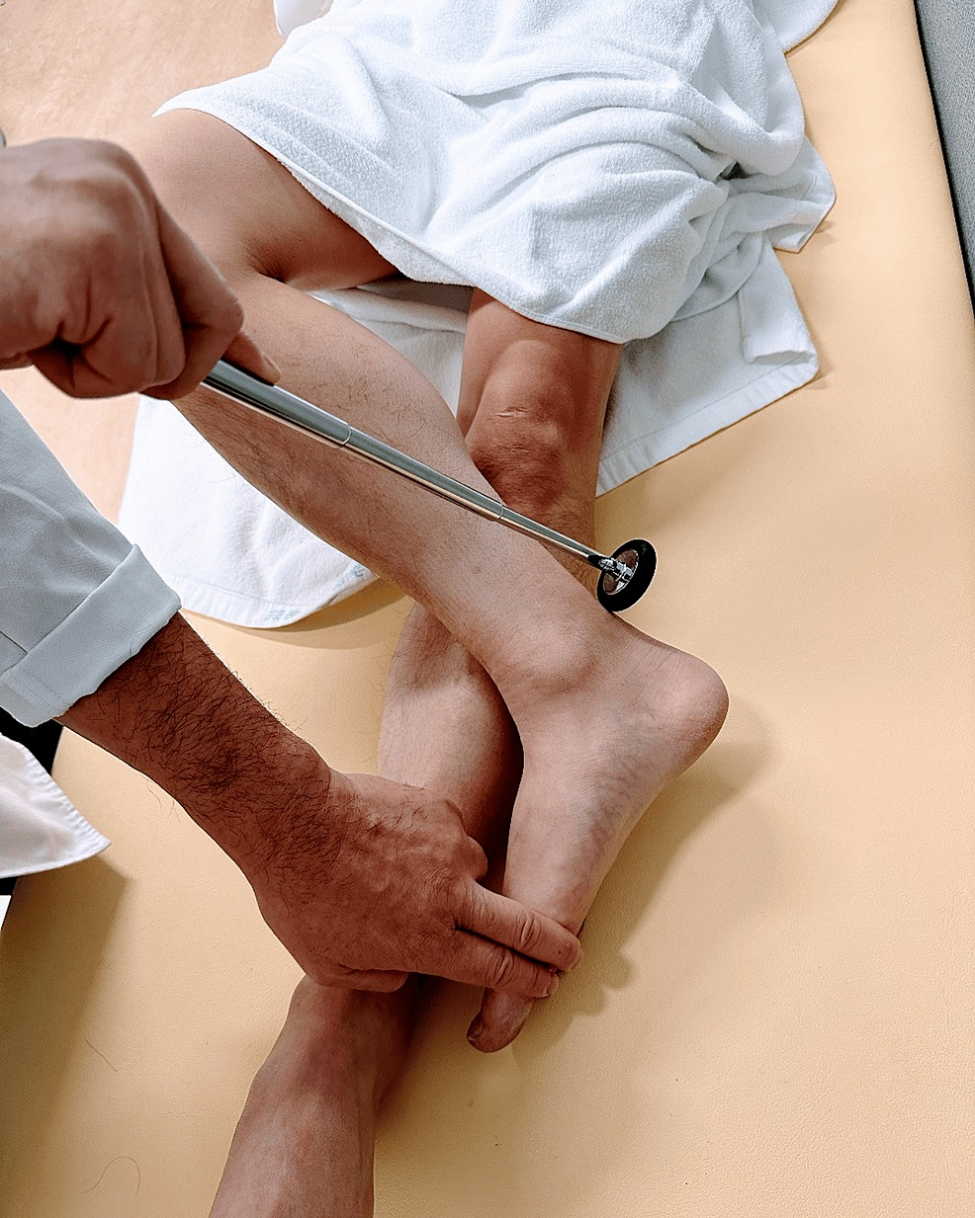
Achilles tendon reflex: supine position (figure four position) One leg is on the other shin or ankle in the same position.

Achilles tendon reflex (ATR) or ankle jerk [3]: The striking point is the Achilles tendons for ATR, and myotome involves S1 and S2. Contraction occurs in the gastrocnemius and coleus muscles via the tibial nerves. In the prone position, the knee is flexed. In the sitting position, the knee is flexed with the hip moderately abducted and externally rotated. In the supine position, the knee is flexed with hip abduction and external rotation (frog-leg position) (Figure 3) or with one leg on the other shin or ankle in the same position (figure four position) (Figure 4).

**Figure 5:**
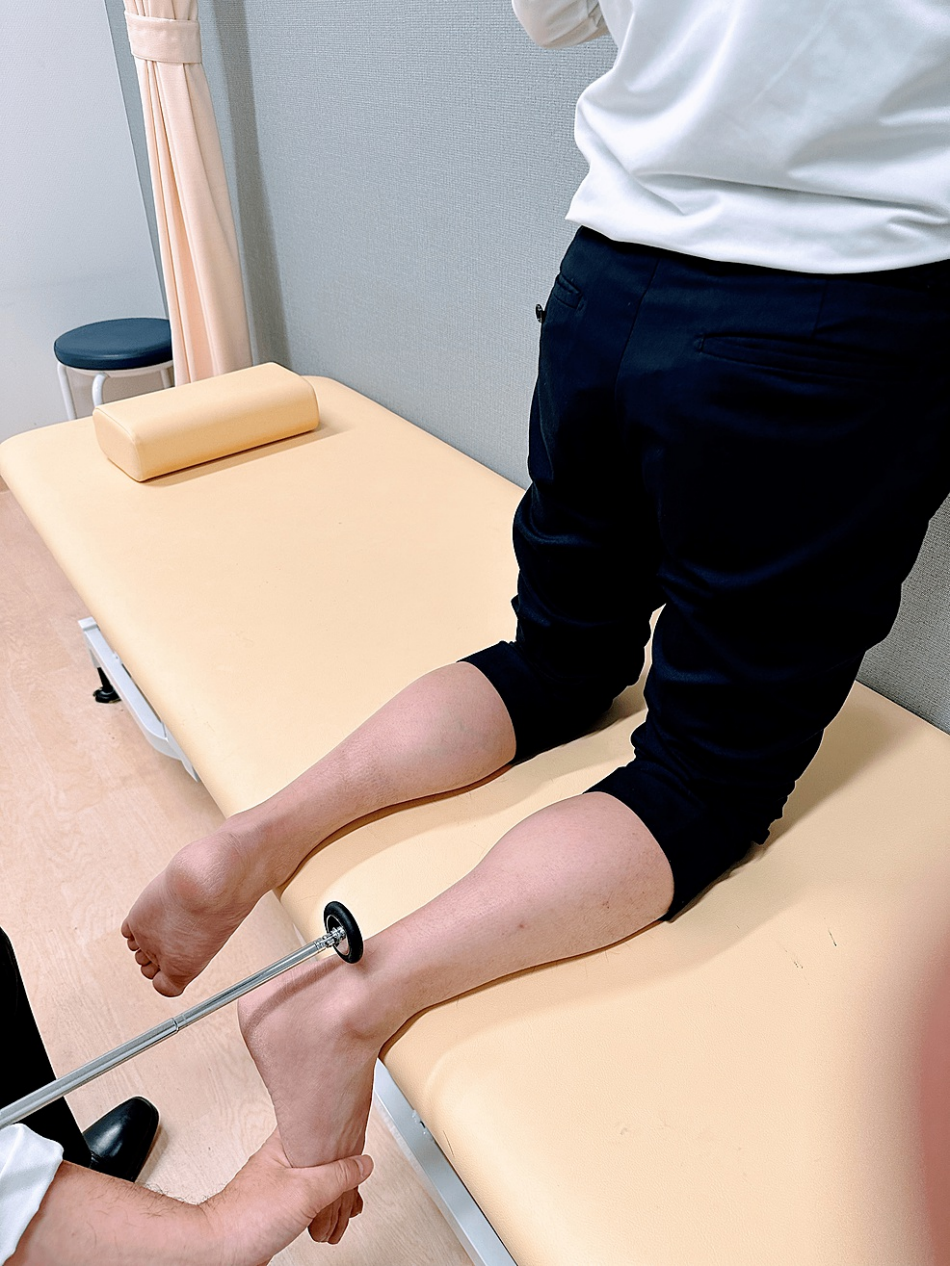
Achilles tendon reflex: kneeling position With the leg hanging from the examination table, the examiner slightly dorsiflexes the ankle joint from the plantar side with one hand and strikes the Achilles tendon with a reflex hammer with the other hand.

In the kneeling position, with the leg hanging from the examination table, the examiner slightly dorsiflexes the ankle joint from the plantar side with one hand and strikes the Achilles tendon with a reflex hammer with the other hand (Figure 5).

Important features of a reflex response include the amount of hammer force required to obtain the contraction, velocity of the contraction, strength of the contraction, duration of the contraction, duration of the relaxation phase, and the response of other muscles that are not tested [3,7]. DTR should only be considered absent if it does not appear after performing an enhancing maneuver.

Jendrassik maneuver: The Jendrassik maneuver is a reinforcement technique for DTR that uses remote muscle contraction [8-10]. The patient attempts to pull the hands apart with fingers hooked together two seconds before the DTR test with or without teeth clenching (Figure 3) [8]. The mechanism by which this effect reinforces the reflex is not well known; however, one theory is that this maneuver causes voluntary upper motor neuron activation, which counters some of the descending inhibition from the brain to the lower motor neuron reflex arc [8-10]. It also prevents conscious inhibition of the reflex by the patient as the patients focus more on the maneuver and less on the examiner [11]. The facilitation by this maneuver only lasts from one to six seconds after the initiation of voluntary contraction [12]. The maximum is only 300 milliseconds [13].

Scale for Evaluating DTR

The National Institute of Neurological Disorders and Stroke (NINDS) provides a standard grading scale [3,7,14]. The British scale is a 5-point scale that adds “+” after the score to distinguish between manual muscle testing scores. This scale distinguishes sustained clonus (5+) from intermittent clonus (4+) [1-3,7]. The scales used are listed in Table 1.

**Table 1:** Assessment scales for deep tendon reflex N/A: not applicable

Neurological Disorders and Stroke Scale	British Scale	Notes
0: Reflex absent	0: Reflex absent	No perceptible response. No reflexes should ever be described as “absent” unless the Jendrassik maneuver has been performed.
1: Reflex small, less than normal, includes a trace response or a response brought out only with reinforcement	1+: Reflex small, less than normal, includes a trace response or a response brought out only with reinforcement	Contraction barely but definitely perceptible
2: Reflex in the lower half of a normal range	2+: Brisk, within the median normal range	Contraction obviously perceptible
3: Reflex in the upper half of a normal range	3+: Reflex enhanced, high normal or hyperreflexia	Vigorous contraction apparent from across the room
4: Reflex enhanced; more than normal; includes clonus if present, which optionally can be noted in an added verbal description of the reflex	4+: Reflex enhanced, more than normal, includes intermittent clonus	Reflex “beats” multiple times at the end of the arc
N/A	5+: Sustained clonus	N/A

No reflexes should ever be described as “absent” unless the Jendrassik maneuver is performed. Therefore, regarding medical education, medical students and residents must be made aware of the reflex enhancement method (Jendrassik method) and the description of “absent” reflex.

Pathological Reflex/Sign

Babinski reflex/sign: To test for the Babinski sign, the instrument is run up the lateral plantar side of the foot from the heel to the lateral toes. If dorsiflexion (upward movement) of the big toe is observed, the Babinski reflex is present. The Babinski sign is considered the result of a decrease in the inhibition of the spinal flexor reflex modulated by the supplementary motor area, indicating a disorder of the central nervous system [15]. The Babinski sign has low sensitivity (50.8%, 95% confidence interval (CI): 41.5-60.1) but high specificity (99%, 95% CI: 97.7-100) for identifying pyramidal tract dysfunction, with an intra-observer reliability of 0.467-0.571 [16]. The presence of the Babinski sign indicates pyramidal tract dysfunction with high specificity. However, because of its low sensitivity, the absence of the Babinski sign does not always indicate pyramidal tract dysfunction. Additional evaluation is required if spinal cord or brain diseases are suspected.

Chaddock reflex/sign: To test for the Chaddock reflex, the dorsolateral aspect of the foot is measured from the posterior portion of the skin immediately beneath the lateral malleolus, anterior to the lateral edge of the foot. If big toe dorsiflexion (upward movement) is observed, the Chaddock reflex is present. The Chaddock sign is a reasonable alternative to the Babinski reflex when the patient has significant withdrawal from plantar stimulation or if the lateral plantar shows skin lesions, wounds, or infections. Infants up to two years of age show positive Babinski or Chaddock signs without neurological problems because of an incompletely myelinated corticospinal tract [17].

Interpretation of DTR in lumbar spine diseases

The key aspects in the assessment of DTR include the diagnosis of the level of impairment in relation to the symptoms identified in the medical interview and the assessment of sensory and muscle strength. To determine whether an increase or decrease in DTR is pathological, it is necessary to evaluate left-right differences, differences between the upper and lower limbs, and the overall balance of the limb.

Hyperactivity of Patellar Tendon Reflex and Achilles Tendon Reflex

Hyperactivity of the PTR and ATR reflexes can be indicative of upper motor neuron involvement, indicating brain, brainstem, or spinal cord pathologies. If patients presenting with lower extremity pain or numbness have enhanced PTR and ATR reflexes, evaluation of spinal cord lesions instead of lumbar spine disease is warranted. These reflex changes may lead to conditions such as spinal cord tumors, thoracic spinal ligament ossification, and thoracic spinal cord hernia [18]. Although bilateral hyperreflexia may be a normal variant, the presence of clonus is pathological and requires comprehensive assessment.

Hypoactivity of PTR and ATR Reflexes

Diminished reflexes indicate disruptions within the reflex arc. Absent reflexes along with sensory deficits in the corresponding nerve distribution indicate afferent arc lesions, potentially involving the nerves or the dorsal horn. The concurrent absence of reflexes, paralysis, fasciculations, and muscle atrophy suggest efferent arc involvement, possibly affecting the anterior horn cells or efferent nerves. Specifically, L3-L4 lesions affect PTR [19,20], whereas S1 lesions influence ATR [21]. The bilateral absence of ATR commonly indicates peripheral neuropathy; however, this sign can also emerge in cauda equina syndrome. Total knee arthroplasty can result in lower PTR compared to the unaffected contralateral knee [22].

Limitations in the interpretation of DTR for lumbar spine diseases

There are several critical pitfalls when interpreting DTR for lumbar spinal diseases (Table 2).

**Table 2:** Indication and pitfalls in the interpretation of DTR for lumbar spine diseases DTR: deep tendon reflex, U/E: upper extremity, L/E: lower extremity, OPLL: ossification of posterior longitudinal ligament, PTR: patellar tendon reflex, ATR: Achilles tendon reflex, TKA: total knee arthroplasty, N/A: not applicable

Findings	Indication	Pitfalls (different diagnosis)
Left-right differences in the DTR of the lower extremities	Suspected lumbar radiculopathy (lumbar nerve root disorder)	Entrapment neuropathy distal to the nerve root (e.g., piriformis syndrome), injury of muscle or tendon
Asymmetrical PTR (decrease one side)	L3 or L4 radiculopathy of the decreased side	Femoral nerve impairment, injury of quadriceps or patellar tendon TKA
Asymmetrical ATR (decrease one side)	S1 radiculopathy of the decreased side	Sciatic nerve or peroneal nerve impairment, Achilles tendon injury
Unilateral lower extremity DTR enhanced	Radiculopathy of the weak side with hyperreflexia	Cerebrospinal disorders
Bilateral lower extremity DTR enhanced	Lumbar diseases unlikely	Cerebrospinal disorders; natural hyperreflexia; in case of normal upper extremity DTR, suspect impairment at the thoracic spine level (e.g., thoracic tumor, OPLL, and hernia)
Bilateral L/E DTR small or absent	Cauda equina disorder, combination of bilateral L3 or L4 radiculopathy, or S1 radiculopathy or cauda equina lesion	Age-related ATR, small or absent peripheral nerve disease (e.g., diabetic neuropathy)
Absent of PTR with normal ATR	Bilateral L3 or L4 radiculopathy	N/A
Absence of PTR with enhanced ATR	Bilateral L3 or L4 radiculopathy in combination with cerebrospinal disorders (e.g., tandem spinal stenosis)	Epiconus syndrome at thoracolumbar lesions
Discrepancies between neurological and imaging findings	Transitional vertebra, furcal nerve, nerve root anomaly	N/A
Lack of symptoms at rest	Gait loading test and standing extension loading test	N/A

Attention should be paid to examiner and patient factors that make the DTR assessment uncertain. When there is a discrepancy between clinical and imaging findings and the level of the lumbosacral nerve root disorder is difficult to diagnose, the presence of a lumbosacral transitional vertebra, nerve root malformation, or furcal nerve should be considered. In addition, the gait loading test and standing extension loading test, which induce lumbosacral neuropathy, may be performed to provide a rationale for diagnosis.

Examiner Factors

The clinical method of evaluation can be subjective or qualitative, leading to subjective conclusions that may differ among examiners based on their experience [23,24]. Improper DTR assessment techniques may alter the findings. Future work needs to find a way to make DTR evaluations more objective.

Patient Factors

Individual factors: There are individual differences in DTR, with some healthy individuals having hyperreflexia and others having hyporeflexia. In addition, reflexes vary throughout the day. It is important to compare both sides simultaneously, with reflex asymmetry considered abnormal [25]. However, a stronger response tends to be observed on the dominant side, which causes non-significant differences; therefore, repeated evaluations are required [26].

Age-related changes in lower extremity DTR: Neurologically normal individuals experience an age-dependent decline in DTR [27,28]. Aging-related reflex decline could be explained by weaker muscle contractions owing to a progressive decrease in the number of muscle fibers [29] or inactivity and atrophy of the muscle that is associated with aging [30]. The other is a reduction in neuronal excitations [31]. Particularly, because ATR is absent in 7%-33% of normal individuals aged >60 years, its evaluation should be consistent with other findings on examination [32-34]. The presence or absence of left-right differences is also important in the evaluation of the pathological significance of DTR. Bilateral loss of the DTR should be differentiated from cauda equina syndrome. In addition, left-right differences in DTR suggest the possible presence of unilateral nerve root compression syndrome on the absent side. To distinguish these changes of pathological significance from normal aging changes, physical findings other than DTR must be considered during diagnosis.

Sex: Reflex responses are generally higher among female subjects than among males [30]. One study using surface electromyography showed that males have a slower patellar reflex than females [3]. Other studies support higher reflex responses among females [26,31], but others do not [35]. Therefore, sex differences in the DTR remain controversial.

Peripheral nerve disease: Diabetic neuropathy (distal symmetric polyneuropathy (DSPN)) causes bilateral decrease or loss of ATR. DSPN is characterized by the symmetrical onset and progression of both subjective symptoms and physical findings centered on the distal portion of the lower extremity. Nerve conduction studies are essential for a definitive diagnosis; however, the diagnostic criteria by the Toronto Diabetic Neuropathy Expert Group are widely used as a simplified diagnostic standard [36]. These criteria include obvious bilateral loss or reduction of the ATR. Thus, the presence of ATR should be considered in the absence of diabetic neuropathy. Risk factors for the development and progression of diabetic neuropathy include poor glycemic control, duration of diabetes, hypertension, lipid abnormalities, smoking cessation, and obesity. The presence of neuropathy should be especially suspected in patients with these conditions [37,38]. Alcoholic neuropathy is thought to be caused by the direct toxicity of alcohol to peripheral nerves due to long-term alcohol use and nutritional disorders such as vitamin deficiencies that often accompany alcohol use. Alcoholic neuropathy is more frequent in heavy drinkers who consume large amounts of alcohol over a long period. A report from the United States found that 25%-60% of alcohol-dependent persons had alcoholic neuropathy [39], which is a chronic, slowly progressive disease that typically takes the form of sensorimotor polyneuropathy with distal dominance in the lower extremities. Tendon reflexes were generally diminished and absent. Alcoholic myelopathy may cause spasticity of the lower extremities and hyperactivity of tendon reflexes, which should be noted. Chemotherapy-induced peripheral neuropathy (CIPN) is one of the most frequent side effects of chemotherapy for cancer [40]. With the aging of the population and increasing survival rate of cancer patients, CIPN is increasingly encountered in daily practice. It is characterized by a glove-and-stocking sensory disturbance that progresses with treatment. Sensory symptoms such as pain, numbness, and tingling are the most common symptoms; however, motor weakness, autonomic dysfunction, and even cranial nerve involvement may occur [40]. Platinum analogs, taxanes, vinca alkaloids, and proteasome inhibitors are the most common causative agents [41]. The neurotoxicity of drugs is dose-dependent, and symptoms become more apparent as treatment progresses. With the progression of symptoms, there is a decrease or loss of deep lower extremity reflexes. Other peripheral neuropathies such as vitamin B12 deficiency, hypothyroidism, and chronic inflammatory demyelinating polyneuropathy can also result in decreased DTRs [42].

Transitional Vertebra and Nerve Root Anomalies Requiring Attention in Neurological Diagnostics

Transitional vertebra: During spine and nerve development, bony factors differentiate first as a vessel for nerve roots, followed by the secondary differentiation of nerve roots according to their vessel [43]. A transitional vertebra is defined as a transverse process of the lowest lumbar vertebra that is fused with the sacrum. Its bony morphology has been classified by Castellvi et al. [44]. In the transitional vertebrae, a segment transition appears secondarily owing to the presence of abnormalities in the differentiation of the bony factors of the spine. Consequently, if the morphology of the transitional vertebra is asymmetrical, the nerve root differentiation is also asymmetrical. It is also highly likely that the differentiation of nerve roots on the left and right sides is asymmetrical owing to differences in the bundling of the upper and lower nerve elements during differentiation. Thus, in cases of nerve root disorders in patients with transitional vertebrae, the impaired nerve root should be identified with consideration of the possibility of asymmetry in the running of the nerve root and its function. Asymmetries in nerve root running and function are present in at least 16% of cases of transitional vertebrae, which are actually morphologically asymmetric [45]. In clinical practice, the function of each nerve root can be determined from neurological deficits that appear in the area innervated by the impaired nerve root. However, the function and running of the nerve root at the transitional intervertebral level are not always typical [46]. There is also a discrepancy in the innervation of the DTR when the L5 vertebra is sacralized or when the S1 vertebra is lumbarized. That is, when the transitional vertebra is located caudal to the fourth lumbar vertebra (L4/Tv), the distribution of the lumbosacral segments deviates from the cephalad. In contrast, when the transitional vertebra is located caudal to the fifth lumbar vertebra (L5/Tv), the distribution of the lumbosacral segments deviates caudally. Furthermore, considerable individual variation has been observed, and in many cases, the anatomical and functional levels of the nerve roots do not coincide. Notably, when the lumbosacral region has a transitional vertebra, its nerve root function is probably abnormal.

Furcal nerves: Furcal nerves are independent spinal nerves that branch across both the lumbosacral and sacral plexus [47]. This spinal nerve has a unique dorsal root ganglion, and its anterior branches branch into the lumbosacral nerve trunk, femoral nerve, and obturator nerve, innervating both the extensor and flexor muscles. The furcal nerve must always be taken into account when considering the diversity of lumbosacral nerve root symptoms. Furcal nerves are classified into six types according to their levels [47]. Clinically, if the bifurcation nerve accompanies the fifth lumbar nerve root, which is the most frequently affected nerve root, symptoms suggestive of fourth lumbar nerve root or first sacral nerve root involvement may also be present.

Congenital dysplasia: Congenital dysplasia of nerve roots is not uncommon [48]. Several types of anomalies have been reported, including two nerve roots branching from the dura mater at the same site with a common sheath (bifurcation anomaly), two nerve roots running together into a single intervertebral foramen (running anomaly), and a traffic branch between the nerve roots (traffic branch). The occurrence of nerve root symptoms in such anomalies is considered atypical. Cannon et al. [49] reported that 2% of surgical cases have nerve root anomalies; they classify them into three types: conjoined nerve roots, anastomotic roots, and transverse roots. Neidre and MacNab’s classification and Kadish and Simmonds’ classification are well-known and useful [9,50]. Nerve root anomalies such as nerve root branching or migration can cause various neurological symptoms. That is, there is a discrepancy between the symptoms assumed at the responsible level and actual clinical symptoms and neurological findings. Congenital dysplasia of the nerve roots should be suspected when nerve root symptoms are atypical.

Gait Loading and Standing Extension Loading Tests

In conditions that present with neurogenic intermittent claudication, such as lumbar spinal stenosis, there are many cases in which subjective symptoms and abnormal neurological findings are not observed at rest, and these findings change with continued walking or standing [51,52]. DTR is typically assessed in an examination room at rest; thus, symptoms that change during walking or standing are likely undetectable [53]. For example, in patients with L3-L4 and L4-L5 spinal stenosis on magnetic resonance imaging, DTR may be normal at rest and may be decreased after walking or standing. A decrease in PTR after continued walking or standing provides objective support for the involvement of L3-L4 at the responsible level. Given that dynamic factors may influence DTR in spinal disorders, this possibility should be considered when findings at rest deviate from imaging findings.

## Conclusions

Despite modern advances in imaging technology, hammer-based assessment of deep lower limb reflexes remains an essential test for lumbar spine diseases. Understanding the basic principles of deep lower limb reflexes, learning the correct technique, and proper interpretation will help in understanding the pathophysiology of lumbar spine diseases. When there is a clinical (physiological or neurological discrepancy) from imaging findings, knowledge of the factors inducing various lumbosacral neurological issues, such as lumbosacral transitional vertebrae, nerve root malformations, furcal nerve, and neurological findings apparent with gait loading or standing, may provide a diagnostic rationale.
